# Adherence to guideline recommendations in the management of pediatric cardiac arrest: a multicentre observational simulation-based study

**DOI:** 10.1097/MEJ.0000000000000923

**Published:** 2022-03-29

**Authors:** Francesco Corazza, Valentina Stritoni, Francesco Martinolli, Marco Daverio, Marco Binotti, Giulia Genoni, Pier Luigi Ingrassia, Marco De Luca, Giordano Palmas, Ilaria Maccora, Anna Chiara Frigo, Liviana Da Dalt, Silvia Bressan

**Affiliations:** a Department of Woman’s and Child’s Health, Division of Paediatric Emergency Medicine, University of Padua; b Department of Woman’s and Child’s Health, Paediatric Intensive Care Unit, University of Padua, Padua; c Neonatal and Paediatric Intensive Care Unit, Maggiore della Carità University Hospital, University of Piemonte Orientale, Novara, Italy; d Centro di Simulazione (CeSi), Centro Professionale Sociosanitario di Lugano, Lugano, Switzerland; e Paediatric Simulation Centre, Meyer Children’s University Hospital; f Department of Health Sciences, University of Florence and Meyer Children’s University Hospital, Florence; g Department of Cardiac, Biostatistics, Epidemiology and Public Health Unit, Thoracic, Vascular Sciences and Public Health, University of Padua, Padua, Italy

**Keywords:** advanced cardiac life support, heart arrest, medical errors, resuscitation, pediatrics, simulation training

## Abstract

**Background and importance:**

Pediatric cardiac arrest is a rare emergency with associated high mortality. Its management is challenging and deviations from guidelines can affect clinical outcomes.

**Objectives:**

To evaluate the adherence to guideline recommendations in the management of a pediatric cardiac arrest scenario by teams of pediatric residents. Secondarily, the association between the use of the Pediatric Advanced Life Support-2015 (PALS-2015) pocket card, and the teams’ adherence to international guidelines, were explored.

**Design, settings and participants:**

Multicentre observational simulation-based study at three Italian University Hospitals in 2018, including PALS-2015 certified pediatric residents in their 3rd–5th year of residency program, divided in teams of three.

**Intervention or exposure:**

Each team conducted a standard nonshockable pediatric cardiac arrest scenario and independently decided whether to use the PALS-2015 pocket card.

**Outcome measure and analysis:**

The primary outcome was the overall number and frequency of individual deviations from the PALS-2015 guidelines, measured by the novel c-DEV15plus score (range 0–15). Secondarily, the performance on the validated Clinical Performance Tool for asystole scenarios, the time to perform resuscitation tasks and cardiopulmonary resuscitation (CPR) quality metrics were compared between the teams that used and did not use the PALS-2015 pocket card.

**Main results:**

Twenty-seven teams (81 residents) were included. Overall, the median number of deviations per scenario was 7 out of 15 [interquartile range (IQR), 6–8]. The most frequent deviations were delays in positioning of a CPR board (92.6%), calling for adrenaline (92.6%), calling for help (88.9%) and incorrect/delayed administration of adrenaline (88.9%). The median Clinical Performance Tool score was 9 out of 13 (IQR, 7–10). The comparison between teams that used (*n* = 13) and did not use (*n* = 14) the PALS-2015 pocket card showed only significantly higher Clinical Performance Tool scores in the former group [9 (IQR 9–10) vs. 7 (IQR 6–8); *P* = 0.002].

**Conclusions:**

Deviations from guidelines, although measured by means of a nonvalidated tool, were frequent in the management of a pediatric cardiac arrest scenario by pediatric residents. The use of the PALS-2015 pocket card was associated with better Clinical Performance Tool scores but was not associated with less deviations or shorter times to resuscitation tasks.

## Introduction

Cardiac arrest is uncommon in pediatrics, but it is associated with significant long-term morbidity, high mortality and relevant socio-economic costs [1,2]. Its management is complex, as several life-saving time-dependent interventions need to be carried out simultaneously. Furthermore, the low frequency of pediatric cardiac arrest makes the acquisition of the necessary resuscitation skills and competence for its management challenging for all providers, especially for doctors in training, who are often the frontline physicians initially managing pediatric emergencies in many settings. The most important international scientific societies periodically update evidence-based guidelines on pediatric advanced life support (PALS), with the purpose of optimizing the management of pediatric cardiac arrest [3]. Nevertheless, deviations from international guidelines during the management of pediatric cardiac arrest have been frequently reported and are associated with poorer clinical outcomes, such as successful Return of Spontaneous Circulation (ROSC) [4].

To facilitate the correct management of pediatric cardiac arrest, scientific societies crafting international guidelines have developed pocket-sized reference cards including flowcharts to guide the course of resuscitation actions. However, the impact of these cognitive aids on resuscitation performance remains largely unknown [5].

The primary aim of this study was to evaluate the adherence to guideline recommendations during the management of a simulated case scenario of in-hospital nonshockable pediatric cardiac arrest by pediatric residents in a multicentre setting. The secondary objective was to explore a possible association between the use of the American Heart Association (AHA) PALS-2015 pocket card [6,7] and the adherence to international guidelines and resuscitation performance, in terms of technical skills.

## Methods

### Study design and participants

This was a prospective observational simulation-based study conducted at three Italian University Hospitals (Padua, Florence and Novara) in 2018. The study was designed according to the ‘Reporting Guidelines for Health Care Simulation Research’ [8]. According to the institutional review boards, the present project qualified for the exempt status.

PALS-2015 certified pediatric residents attending their 3rd–5th year of the residency program were included. We excluded residents who were not available for the simulation sessions because of maternity/sick/personal leave or training abroad. All participants provided written informed consent for study participation and video recording of the simulation sessions.

### Study procedures

Teams of three residents, stratified by the recruiting site, were formed by the research team based on the random selection of participants. One of the team members was designated to be the team leader at the team discretion, before the scenario. The case scenario was unknown to the participants before starting the simulation. Each team was introduced to the scenario through a standardized briefing and a video, where an actress, in the role of the simulated patient’s mother, gave basic history information. Each team then conducted the same 10-min simulated scenario of nonshockable pediatric cardiac arrest (Supplemental digital content 1, http://links.lww.com/EJEM/A329) with the help of a confederate nurse who acted in a standardized manner, following a predetermined script. A nonshockable rhythm was chosen for the scenario because it is the most frequent rhythm in pediatric cardiac arrest [2]. The simulation sessions were organized off-site at each participating institution, in rooms set up to reproduce the environment of the pediatric emergency department shock room. The same scenario, equipment and high-fidelity mannequin (HAL S 3005 Gaumard) were used at all sites.

The AHA-PALS-2015 pocket card was available for use for participants in each scenario. This paper-based cognitive aid displays the AHA-PALS-2015 algorithms and the sequence/content of recommended interventions for pediatric emergencies [6,7]. Each team leader made an independent decision on whether to use it.

All scenarios were video recorded. After the simulation, each team conducted a 20-min debriefing (plus/delta/solution method) and each participant completed a questionnaire about demographics and previous experience in simulation and real pediatric cardiac arrest events. At the end of the debriefing, all participants were asked not to communicate details of the scenario to other colleagues. All video recordings were subsequently analyzed and scored by two independent and previously trained reviewers. Disagreements between reviewers were resolved through discussion with a third expert reviewer.

## Outcomes

### Primary outcome

The primary outcome was the overall number of deviations and the frequency of individual deviations from the AHA-PALS-2015 guidelines [7]. Deviations were measured by a novel 15-item scoring system named circulation-deviations 15 plus (c-DEV15plus), developed to evaluate the omissions/errors/delays about recommended resuscitation tasks. This score was previously used in a pilot study [9]. Each item identifies a specific correct resuscitation task, and it can be scored as 0 (if the action is well and timely performed) or 1 (if the action is not performed, performed late/incorrectly, deviating from guideline recommendations). This tool was created, based on the consensus of our research team, using previously published guidelines/checklists/scoring systems [4,7–12] and ranges from 0, if no deviation is registered, to a maximum of 15 (Table 1).

**Table 1:** C-DEV15plus score

c-DEV15plus items for non-shockable pediatric cardiac arrest simulation scenario
1. CPR started within 30 s (s) from recognition of pulseless state
2. CPR board/rigid surface positioned underneath the manikin within 60 s from recognition of pulseless state
3. Compression/ventilation ratio 15:2
4. Help called (hospital emergency response system activated) within 60 s from recognition of pulseless state
5. Compressors switched more than once during CPR
6. EKG-monitoring started within 60 s from recognition of pulseless state
7. IV/IO access called within 60 s from recognition of pulseless state
8. First adrenaline called within 30 s from recognition of pulseless state
9. First adrenaline administered at the correct dose and dilution^a^ and by the correct route (IV or IO), followed by a normal saline flush, while compressions are being performed, within 180 s (3 min) from recognition of pulseless state
10. Second adrenaline called between 3 and 5 min from the first administration of adrenaline
11. Second adrenaline administered at the correct dose and dilution ^a^ and by the correct route, followed by a normal saline flush, while compressions are being performed, within 5 min from the first adrenaline
12. Blood gas called during cardiac arrest
13. Reversible causes treated
14. Shock not administered
15. Medications other than adrenaline (e.g. amiodarone, lidocaine, atropine) not administered^b^

CPR, cardiopulmonary resuscitation; EKG, electrocardiogram; IO, intraosseous; IV, intravenous.

a Correct dose of adrenaline is defined as 0.01 mg/kg (or a deviation from the correct weight dose of less than 10%); correct dilution of adrenaline is defined as 1:10.000 (0.1 mg/ml).

b Administration of medications to treat identified reversible causes is not considered in this item.

### Secondary outcomes

#### Secondary outcomes were:

(1) Teams’ resuscitation performance (technical skills), measured by the Clinical Performance Tool [10]. This is a validated tool divided in different sections based on the type of pediatric emergency scenario. We used the scoring instrument for the asystole scenario, which includes seven items. The overall score ranges from 0 to 13; a higher value corresponds to better performance (further details about the Clinical Performance Tool are available in Supplemental digital content 2, http://links.lww.com/EJEM/A329).
(2) Time to resuscitation actions, defined as the time (in seconds from the beginning of the scenario) to the performance of critical resuscitation actions.
(3) Cardiopulmonary resuscitation (CPR) quality metrics, as measured by the internal software of the mannequin (Gaumard), are evaluated according to the AHA standards [3]. In particular, the no-flow fraction was defined as the percentage of time of the total resuscitation period when chest compressions were not performed; the chest compressions rate (mean number of compressions per minute) and depth (mean value in centimeters), and chest wall recoil (percentage of compressions where full chest recoil was achieved) were also recorded by the software.

### Statistical analysis

Results for the entire cohort of participating teams and stratified by AHA-PALS-2015 pocket card utilization, are expressed as medians and interquartile range (IQR) in the case of quantitative variables, and with counts and percentages for categorical ones. The normality of quantitative variables was inspected through a Q-Q plot and tested with the Shapiro-Wilk test. Fisher’s exact test was used to compare qualitative variables between teams using and not using the AHA-PALS-2015 pocket card. The difference between the two groups was estimated with 95% confidence intervals (CI) calculated with the binomial method. Wilcoxon rank-sum test was applied for inter-group comparison of quantitative variables with the median difference between the two groups estimated by 95% CI calculated with the Hodges-Lehmann method. This is because a nonparametric method to test the hypothesis of difference between the groups was used. Agreement between the two video reviewers was estimated with the Lin’s Concordance Correlation Coefficient (CCC) [13,14] and the 95% CI was obtained with 2000 bootstrap resampling. The statistical significance was declared for *P*< 0.05. Analyses were performed by an external statistician (A.C.F.) using SAS 9.4 (SAS Institute Inc., Cary, North Carolina, USA) for Windows.

## Results

### Characteristics of participants

**Fig. 1:**
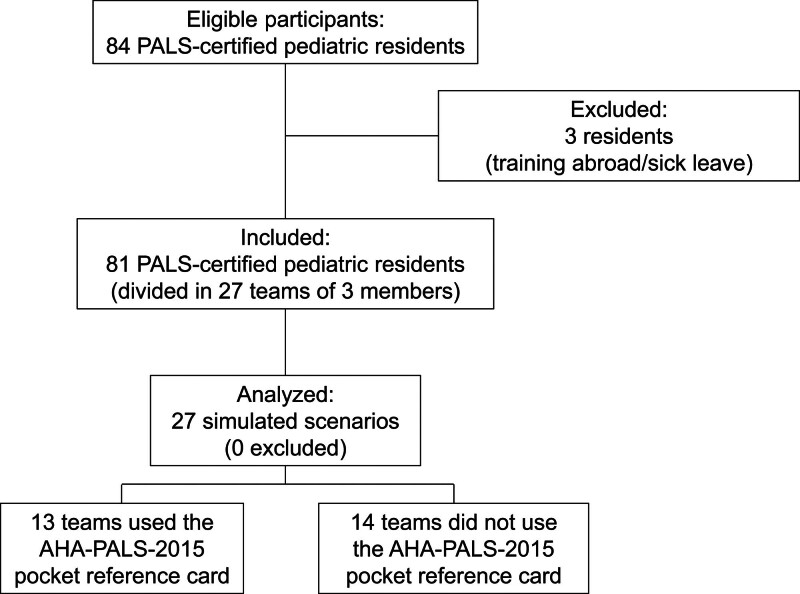
Study flow diagram. AHA, American Heart Association; PALS, Pediatric Advanced Life Support.

Eighty-one pediatric residents, divided into 27 teams of three members, were included in the study (Fig. 1). The majority of participants were attending their 5th year of residency program (38.0%), obtained their PALS certification in the previous two years (67.1%) and experienced a real-life resuscitation (62.0%) (Table 2). Thirteen teams (48%) used the AHA-PALS-2015 pocket card, based on the team leader’s independent choice. The characteristics of participants of the teams that used and did not use the AHA-PALS-2015 pocket card were similar (Table 2).

**Table 2:** Participant characteristics

	Overall *n* = 79^a^	No AHA-PALS pocket reference card group *n* = 41^b^	AHA-PALS pocket reference card group *n* = 38^b^	*P*value^c^
Year of residency program, *n* (%)				
3rd year of residency program	23 (29.1)	13 (31.7)	10 (26.3)	0.782
4th year of residency program	26 (32.9)	12 (29.3)	14 (36.8)	
5th year of residency program	30 (38.0)	16 (39.0)	14 (36.8)	
Time since PALS certification, *n* (%)				
Less than 1 year since PALS certification	30 (38.0)	16 (39.0)	14 (36.8)	0.750
From 1–2 years since PALS certification	23 (29.1)	10 (24.4)	13 (34.2)	
From 2–3 years since PALS certification	20 (25.3)	12 (29.3)	8 (21.1)	
More than 3 years since PALS certification	6 (7.6)	3 (7.3)	3 (7.9)	
Previous simulation training, *n* (%)	68 (86.1)	35 (85.4)	33 (86.8)	1.000
Previous real-life resuscitation experience, *n* (%)	49 (62.0)	26 (63.4)	23 (60.5)	0.820

%, percentage of participants; AHA, American Heart Association; n, number of participants; PALS, pediatric advanced life support.

a Two participant data missing.

b One participant data missing.

c Comparison between the group that used and did not use the AHA-PALS-2015 pocket reference card.

### Primary outcome

Overall, the median number of deviations from the AHA-PALS-2015 guidelines, calculated using the c-DEV15plus score for each of the 27 teams, was 7 out of 15 (IQR 6–8). The most frequent deviations included delayed positioning of a CPR board (92.6%), delayed call for first adrenaline (92.6%), delayed call for help (88.9%), incorrect/delayed administration of adrenaline (88.9%), delayed call for second adrenaline (77.8%), lack of treatment of reversible causes (63%) and lack of compressor switch (51.9%) (Table 3, Supplemental digital content 3, http://links.lww.com/EJEM/A329).

**Table 3:** Deviations from guideline recommendations as per cDEV15plus score individual items

	Total *n* = 27^c^	No AHA-PALS pocket reference card *n* = 14	AHA-PALS pocket reference card *n* = 13	AHA-PALS vs. No AHA-PALS (95% CI)	*P*^d^value
CPR started within 30 s from recognition of pulseless state, *n* (%)	26 (96.3)	14 (100.0)	12 (92.3)	−7.8 (−42.8 to 29.5)	0.482
Position CPR board underneath the manikin within 60 s from recognition of pulseless state, *n* (%)	2 (7.4)	0 (0.0)	2 (15.4)	15.4 (−22.6 to 49.2)	0.222
Correct compression:ventilation ratio, *n* (%)	23 (85.2)	10 (71.4)	13 (100.0)	28.6 (−8.1 to 61.3)	0.098
Call for emergency team help within 60 s from recognition of pulseless state, *n* (%)	3 (11.1)	2 (14.3)	1 (7.7)	−6.6 (−42.8 to 29.5)	1.000
Compressors switched more than once during CPR, *n* (%)	13 (48.1)	7 (50.0)	6 (46.2)	−3.8 (−41.6 to 34.5)	1.000
EKG-monitoring started within 60 s from recognition of pulseless state, *n* (%)	22 (81.5)	11 (78.6)	11 (84.6)	6.4 (−29.5 to 42.8)	1.000
IV/IO access called within 60 s from recognition of pulseless state, *n* (%)	17 (63.0)	10 (71.4)	7 (53.8)	−17.6 (−22.2 to 52.3)	0.440
First adrenaline called within 30 s from recognition of pulseless state, *n* (%)	2 (7.4)	1 (7.1)	1 (7.7)	0.6 (−36.3 to 36.3)	1.000
First adrenaline administered at the correct dose and dilution^a^ and by the correct route (IV or IO), followed by a normal saline flush, while compressions are being performed, within 180 s (3 min) from recognition of pulseless state, *n* (%)	3 (11.1)	1 (7.1)	2 (15.4)	8.2 (−29.5 to 42.8)	0.596
Second adrenaline called between 3 and 5 min from the first administration of adrenaline, *n* (%)	6 (22.2)	2 (14.3)	4 (30.8)	16.5 (−22.6 to 49.2)	0.385
Second adrenaline administered at the correct dose and dilution^a^ and by the correct route, followed by a normal saline flush, while compressions are being performed, within 5 min from the first adrenaline, *n* (%)	8 (29.6)	1 (7.1)	7 (53.8)	46.7 (7.3–75.2)	**0.013**
Call for blood gas, *n* (%)	21 (77.8)	10 (71.4)	11 (84.6)	13.2 (−22.6 to 49.2)	0.648
Treatment of reversible causes, *n* (%)	10 (37.0)	5 (35.7)	5 (38.5)	2.7 (−35.9 to 39.3)	1.000
Shock not administered *n* (%)	26 (96.3)	13 (92.9)	13 (100.0)	7.1 (−29.5 to 42.8;)	1.000
Medications other than adrenaline (e.g. amiodarone, lidocaine, atropine)^b^ not administered, *n* (%)	26 (96.3)	13 (92.8)	13 (100.0)	7.1 (−29.5 to 42.8)	1.000

%, percentage of teams; AHA, American Heart Association; c-DEV15plus, circulation-deviations 15 plus; CI, confidence interval; CPR, cardiopulmonary resuscitation; EKG, electrocardiogram; IO, intraosseous; IV, intravenous, n, number of teams; PALS, Pediatric Advanced Life Support.

a Correct dose of adrenaline is defined as 0.01 mg/kg (or a deviation from the correct weight dose of less than 10); correct dilution of adrenaline is defined as 0.1 mg/ml (1:10.000).

b Administration of medications to treat identified reversible causes is not considered in this item.

c Comparison between the group that used and did not use the AHA-PALS-2015 pocket reference card.

d The absolute number and percentages in the table refers to the actions correctly performed according to the definition of each checklist item. The percentage frequency of deviations can be calculated as complement of 100%.

The c-DEV15plus score did not statistically differ between teams who used (*n* = 13) and did not use (*n* = 14) the AHA-PALS-2015 pocket card [median 7 (IQR 5–8) vs. 8 (IQR 7–9), *P* = 0.078; median of difference = −1, 95% CI, −2 to 0) (Table 3). The inter-rater reliability between the two video reviewers was good (CCC = 0.887; 95% CI, 0.763–0.971) [14]. Further details about adherence to individual resuscitation tasks not included in the c-DEV15plus score are available in Supplemental digital content 3, http://links.lww.com/EJEM/A329.

### Secondary outcomes

The evaluation of the teams’ resuscitation performance showed an overall Clinical Performance Tool median score of 9 out of 13 (IQR 7–10). Times to perform individual resuscitation tasks are reported in Table 4. Long times to action were observed overall for calling for help, establishing an IV/IO access and administering adrenaline. CPR quality metrics showed that 85% of teams had a mean compression rate that was lower than the recommended 100/min, and a mean compression depth that was below/above the recommended 50–60 mm. Eighteen teams (66.7%) had a complete chest recoil in less than 50% of compressions. The no-flow fraction was less than 20% in 35% of teams.

**Table 4:** Times to perform individual resuscitation tasks and quality of cardiopulmonary resuscitation

	Total *n* = 27		No AHA-PALS pocket reference card use group *n* = 14		AHA-PALS pocket reference card use group *n* = 13		AHA-PALS vs No AHA-PALS pocket reference card use group (95% CI)	*P*^a^value
	*n*	Mdn (IQR)/%	*n*	Mdn (IQR)/%	*n*	Mdn (IQR)/%		
Time to resuscitation tasks								
Time to check pulse (s)	26	27.0 (23.0–38.0)	13	33.0 (25.0–38.0)	13	26.0 (14.0–35.0)	−10 (−21 to 4)	0.182
Time to start CC (s)	27	51.0 (33.0–75.0)	14	52.5 (38.0–91.0)	13	45.0 (23.0–72.0)	−10 (−31 to 15)	0.356
Time to start EKG monitor (s)	27	57.0 (40.0–86.0)	14	44.5 (35.0–60.0)	13	73.0 (52.0–91.0)	21.5 (−4 to 43)	0.094
Time to call for help (s)	20	192.5 (163.0–323.5)	10	177.0 (157.0–355.0)	10	233.0 (169.0–292.0)	32.5 (−91 to 116)	0.623
Time to start bag mask ventilation	27	70.0 (50.0–87.0)	14	73.0 (63.0–87.0)	13	63.0 (44.0–82.0)	−10 (−29 to 11)	0.320
Time to establish IV/IO line (s)	27	190.0 (130.0–246.0)	14	196.0 (130.0–284.0)	13	185.0 (140.0–245.0)	−16.5 (−97 to 55)	0.452
Time to administer adrenaline (s)	24	264.5 (214.0–293.5)	11	274.0 (213.0–360.0)	13	256.0 (215.0–272.0)	−43 (−107 to 18)	0.247
Time to repeat adrenaline (s)	19	376.0 (298.0–445.0)	7	334.0 (270.0–485.0)	12	387.0 (345.5–442.5)	40.5 (−103 to 142)	0.422
CPR quality metrics								
Mean CC rate (compressions/min)	27	85.0 (67.0–96.0)	14	85.5 (69.0–96.0)	13	79.0 (66.0–99.0)	1.5 (−18.0 to 16.0)	0.981
Mean CC depth (cm)	27	6.10 (4.60–7.00)	14	5.50 (4.40–6.30)	13	6.60 (6.00–7.60)	1.3 (0.0–2.6)	0.052
Complete chest recoil (% of CC)	27	45.0 (35.0–54.0)	14	46.5 (41.0–56.0)	13	44.0 (34.0–48.0)	−6.5 (−18.0 to 5.0)	0.308
No-flow fraction (% of time)	26	0.24 (0.18–0.28)	13	0.23 (0.14–0.29)	13	0.24 (0.18–0.27)	0.0 (−0.09 to 0.06)	0.959
Correct CC rate (100–120/min), (% of teams )	4	14.8	1	7.1	3	23.1	15.9 (−22.6 to 49.2)	0.326
Correct CC depth (5–6 mm), (% of teams)	4	14.8	2	14.3	2	15.4	1.1 (−36.3 to 36.3)	1.000
Complete chest recoil in more than 50% of CCs (% of teams)	9	33.3	6	42.9	3	23.1	−19.8 (−55.2 to 17.3)	0.420
No-flow fraction<20% (% of teams)	9	34.6	5	38.5	4	30.8	−7.7 (−46.8 to 33.3)	1.000

%, percentage; AHA, American Heart Association; CC, chest compressions; CI, confidence interval; EKG, electrocardiogram; IO, intraosseous; IQR, interquartile range; IV, intravenous; Mdn, median; n, number of teams; PALS, pediatric advanced life support.

a Comparison between group that used the AHA-PALS pocket reference card and the group that did not use it.

When comparing the group who used the AHA-PALS-2015 pocket card with the group that did not use it, the median Clinical Performance Tool score was higher in the former (median 9, IQR 9–10 versus median 7, IQR 6–8; *P* = 0.002; median of difference = 2, 95% CI, 1–3), while the times to perform individual resuscitation tasks and the CPR quality metrics were not different (Table 4).

## Discussion

This observational multicentre simulation-based study showed a high number of deviations from international guidelines during the management of a nonshockable pediatric cardiac arrest simulated scenario by pediatric residents. Although explorative with this regard, the use of the AHA-PALS-2015 paper-based cognitive aid was not associated with a lower number of deviations, shorter times to perform resuscitation tasks or better quality of CPR, whereas the overall team performance, as measured by the validated Clinical Performance Tool, was higher in the teams that used the AHA-PALS-2015 pocket card. Given that all outcomes used assessed aspects of clinical performance, the clinical meaning of the isolated significant higher Clinical Performance Tool score in the group using the cognitive aid remains unclear and seems to be related to the characteristics of the tools rather than to an actual overall superior management. As a matter of fact, the number, type and scoring of the resuscitation tasks assessed differences between the tools used. In addition, the assessment of adrenaline administration is less granular in the Clinical Performance Tool, which does not include details on the correct administration modalities (dosing/dilution/route). This is because, while the Clinical Performance Tool primarily aims to assess technical skills, the c-DEV15plus score includes a more comprehensive list of deviations leading to ineffective/potentially harmful interventions during the resuscitation of a nonshockable pediatric cardiac arrest.

Similar to our results, a few previous simulation-based studies [15–19], although single center and focusing more on shockable pediatric cardiac arrest, found that deviations from guidelines occur often, especially in checking the pulse, starting CPR, analyzing the rhythm, defibrillating and calling for adrenaline. These are all clinically relevant deviations, as reported by Wolfe *et al*., [4]. Among all the in-hospital pediatric cardiac arrest real events reported, 17% had at least one deviation documented, and any error/delay in processes regarding medications/defibrillation/vascular access/chest compressions were significantly associated with a lower probability to achieve ROSC [4].

Consistently with our findings, other studies reported that chest compressions fraction, leaning, rate and depth were frequently not adequate according to AHA standards during pediatric resuscitations [20]. To optimize CPR quality, different studies have demonstrated that CPR feedback devices appear to improve the quality of chest compressions [21,22]. Furthermore, the role of the CPR coach has proven to be effective to increase the quality of CPR during pediatric cardiac arrest scenarios [23]. Hence, these strategies have been considered reasonable in the most recent international guidelines [3].

In the present study, less than 50% of the teams elected to use the AHA-PALS-2015 pocket card. The reasons why participants, who all had access to the AHA-PALS-2015 pocket card during the scenario, elected whether or not to use this cognitive aid were not systematically investigated through a structured questionnaire. It emerged, however, during the debriefing sessions that participants were so engaged in the management of the scenario that they did not think of using the available cognitive aid, rather than deliberately choosing not to use it. Two different simulation-based observational studies reported a higher percentage of use (approximately 85%) of cognitive aids, displaying AHA-PALS-2015 algorithms, by teams of pediatric residents during simulated pediatric cardiac arrest scenarios [17,18]. Interestingly, in one study 26% of participants who used a cognitive aid chose the incorrect algorithm resulting in inappropriate management [17] and in the other study, only 3.4% of participating teams were prompted by the use of the cognitive aid to initiate CPR [18]. Similarly, the present study found that the AHA-PALS-2015 paper-based cognitive aid use was not associated with a lower number of deviations from guidelines recommendations. Although the reasons for this finding should be better investigated through quantitative randomized controlled trial/qualitative research, an interactive cognitive aid developed based on the users’ needs may prove to be more effective to increase adherence to guideline recommendations.

To reduce the number of deviations from international guidelines, different research teams have recently developed cognitive aids that could support health providers in the management of both adult and pediatric cardiac arrest scenarios, obtaining inconsistent results [9,12,24–27]. Even telemedicine has been assessed as a tool to support the management of resuscitation through real-time audio-video communication with a remote expert [28]. However, the heterogeneity of the study designs, methods, participants, types of intervention/comparator and outcome measures does not allow to conclude in favor or against the use of a specific cognitive aid for the management of cardiac arrest.

Based on the results of the present study and the suggestions provided by participants, our research team developed a tablet app to optimize the management of pediatric cardiac arrest and tested it in a pilot study [9]. The effectiveness of this cognitive support tool in reducing deviations from AHA guidelines is currently under evaluation in a simulation-based randomized controlled trial [29].

The present study adds to the existing literature by providing results on deviations from guideline recommendations in a multicentre setting from a European country and explorative results on the association between the use of a cognitive aid and pediatric cardiac arrest management. There is currently a gap in knowledge on the effectiveness of cognitive aids in improving adherence to guideline recommendations in pediatrics. The only two pediatric studies assessing cognitive aids in pediatric cardiac arrest simulated scenarios compared two different types of cognitive aids, without including a control group that did not use any cognitive support tool [24,25]. As recently highlighted by a systematic review [5], there are no studies assessing the effectiveness of cognitive aids on the management of real-life pediatric cardiac arrest cases. Future high-quality randomized controlled trials will help clarify the effect of cognitive aids in improving adherence to guidelines recommendations.

This study presents limitations. First, the observational nature of the study and the participants’ independent choice for using or not using the AHA-PALS-2015 pocket card did not allow to control for possible selection bias or other factors that may have influenced the results of the study outcomes. However, in the absence of a difference between the study groups in their baseline characteristics, this seems less of a concern. Certainly, the small sample size limited the possibility to detect significant differences in the study outcome, for which the study was not powered. Second, only pediatric residents were included in this study. This population is not totally representative of all clinicians that could assist a victim of in-hospital pediatric cardiac arrest. However, doctors in training are more willing to participate in simulation-based education-related projects and, for this reason, they represent the study population of most simulation-based studies on cardiac arrest [15–18,24–27]. Future studies should include nurses, attendings and residents from other medical programs that are involved in emergency codes. Third, although the c-DEV15plus score was used in a previous study from our research team [9], this score has not been previously validated. We also used, however, the validated Clinical Performance Tool, although focusing on technical skills only. Fourth, while simulation-based studies have the intrinsic limitation to evaluate simulated scenarios and not real events, simulation-based research is a diffusely accepted method to evaluate emergency medicine management interventions [30]. Finally, this study was conducted with the AHA-PALS-2015 pocket card version, before 2020 guidelines were published, and results might have been different if the updated 2020 guidelines and pocket card version had been used [3].

### Conclusion

Deviations from guidelines, although measured by means of a nonvalidated tool, were frequent in the management of a simulated pediatric cardiac arrest scenario by pediatric residents. The use of the AHA-PALS-2015 pocket card appeared to be associated with better Clinical Performance Tool scores, but was not associated with less deviations, shorter times to perform resuscitation tasks and better CPR quality metrics.

## Acknowledgements

The authors thank Dr. Marta Arpone for her critical review of the manuscript, Dr. Martin Diogo for the organization of the simulation sessions, and all pediatric residents who took part into this study.

S.B., V.S., F.M., M.D. and L.D.D. conceptualized and designed the study. V.S., F.M., M.D., L.D.D., S.B., M.B., G.G., P.L.I., M.D.L., G.P., I.M., and F.C. conducted the simulation sessions. A.C.F. performed statistical analyses. F.C. and S.B. wrote the first draft of the article. V.S. and F.C. contributed equally to the article. All the authors refined and approved the final version of the article.

### Conflicts of interest

There are no conflicts of interest.

## Supplementary Material


