# Supplementary material for: Adherence to guideline recommendations in the management of pediatric cardiac arrest: a multicentre observational simulation-based study
Source: Eur J Emerg Med. 2022 Mar 29;29(4):271–8. doi: 10.1097/MEJ.0000000000000923 (PMC10878464; doi:10.1097/MEJ.0000000000000923)
Supplement: Supplementary file 1 [file ejem-29-271-s001.pdf]

Title: **Adherence to guideline recommendations in the management of pediatric cardiac arrest: a multicenter observational simulation-based study**

Authors: Francesco Corazza and Valentina Stritoni, Francesco Martinolli, Marco Daverio, Marco Binotti, Giulia Genoni, Pier Luigi Ingrassia, Marco De Luca, Giordano Palmas, Ilaria Maccora, Anna Chiara Frigo, Liviana Da Dalt, Silvia Bressan.

Supplemental Digital Content 1. Simulation scenario

Case scenario: a case of non-shockable pediatric cardiac arrest (1 minute of pulseless electrical activity followed by asystole).

Team: Three pediatric residents and one confederate nurse.

Setting: off-site, in a room set reproducing the environment and the equipment of a pediatric emergency department shock room.

Presentation to participants: in a video an actress played the mother's role and provided to participants the following information about the case.

- Introduction: A 5-year-old child (20 kg weight) is brought to the Emergency Department by his mother; she refers that the child was playing in a playground with other children and suddenly he fell down and lost consciousness.
- History information: Sign/Symptoms: he suddenly fell while he was playing; Allergies: not known; Medications: none; Past history: previously healthy child; Last meal: he took a snack two hours before (a yogurt and a juice); Events: he was playing with other children.

Return of Spontaneous Circulation was obtained if cardiopulmonary resuscitation was performed and two epinephrine were administered with correct timing (second dose after at least three minutes from the first one), dilution (0.1 mg/ml with normal saline), route of administration (intravenously) and dosage (0.2 mg of epinephrine).

The scenario lasted 10 minutes.

Title: **Adherence to guideline recommendations in the management of pediatric cardiac arrest: a multicenter observational simulation-based study**

Authors: Francesco Corazza and Valentina Stritoni, Francesco Martinolli, Marco Daverio, Marco Binotti, Giulia Genoni, Pier Luigi Ingrassia, Marco De Luca, Giordano Palmas, Ilaria Maccora, Anna Chiara Frigo, Liviana Da Dalt, Silvia Bressan.

Supplemental Digital Content 2. Clinical Performance Tool

Scoring system for asystole scenario (by Levy A, Donoghue A, Bailey B, Thompson N, Jamoulle O, Gagnon R, Gravel J: External validation of scoring instruments for evaluating pediatric resuscitation. Simul Healthc 2014;9(6):360–9) [12].

	0 point	1 point	2 points
Pulse check	• Not done	• >30 s<br>• Peripheral pulse<br>• After CPR started or epinephrine given	• <30 s and in sequence
CPR	• Not done	• Done without pulse check<br>• Done after epinephrine given<br>• >30 s after pulselessness recognized	• <30 s after pulselessness recognized and before epinephrine
ECG	• Not done	• Done without clinical assessment of circulation<br>• Done before CPR if pulseness recognized<br>• Done after epinephrine<br>• >60 s	• Done after CPR started for pulselessness and before other therapy
IV/IO access	• Not done<br>• Only done once need for IV med recognized	• IV instead of IO<br>• >60 s	• IO in < 60 s
Epinephrine	• Not done	• Called for without pulse check<br>• Called for without CPR<br>• Called for without via ETT<br>• >30 s after pulselessness recognized	• Called for after pulse check and CPR within 30 s of pulselessness recognition
Pulse recheck after ROSC	• Not done (includes ROSC never achieved)	• 30 s after ROSC<br>• Peripheral pulse check	• Central pulse checked within 30 s of ROSC
Defibrillation	• Called for	• Never called for	

Abbreviations: CPR: cardiopulmonary resuscitation, ECG: electrocardiography, ETT: endotracheal tube; IV: intravenous, IO: intraosseous, med: medicine, ROSC: return of spontaneous circulation, s: seconds.

Title: **Adherence to guideline recommendations in the management of pediatric cardiac arrest: a multicenter observational simulation-based study**

Authors: Francesco Corazza and Valentina Stritoni, Francesco Martinolli, Marco Daverio, Marco Binotti, Giulia Genoni, Pier Luigi Ingrassia, Marco De Luca, Giordano Palmas, Ilaria Maccora, Anna Chiara Frigo, Liviana Da Dalt, Silvia Bressan.

Supplemental Digital Content 3. Adherence to individual resuscitation tasks

	**Total n= 27**	**No AHA-PALS pocket reference card n = 14**	**AHA-PALS pocket reference card n = 13**	**AHA-PALS vs No AHA-PALS pocket reference card use (95% CI)**	***p*^ value**
Pulse check, n (%)	26 (96.3)	13 (92.9)	13 (100.0)	7.1(-29.5; 42.8)	1.000
Pulse checked within 30 s from the beginning of the scenario, n (%)	12 (44.4)	5 (35.7)	7 (53.8)	18.1(-21.6; 53.5)	0.449
Start chest compressions, n (%)	27 (100)	14 (100.0)	13 (100.0)		NA
Position CPR board underneath the patient, n (%)	7 (25.9)	2 (14.3)	5 (38.5)	24.2 (-15.4; 56.4)	0.209
Call for help, n (%)	20 (74.1)	10 (71.4)	10 (76.9)	5.5 (-29.6; 42.8)	1.000
Start of bag mask ventilation, n (%)	27 (100)	14 (100)	13 (100)		NA
Administration of epinephrine, n (%)	24 (88.9)	11 (78.6)	13 (100.0)	21.4 (-15.4; 55.4)	0.222
Administration of second epinephrine, n (%)	19 (70.4)	7 (50.0)	12 (92.3)	42.3 (7.2; 72.6)	**0.033**
Advanced management of airway, n (%)	5 (18.5)	4 (28.6)	1 (7.7)	-20.9 (-55.4; 15.4)	0.326
Search for reversible causes, n (%)	8 (29.6)	2 (14.3)	6 (46.2)	31.9(-8.0; 63.4)	0.103

Abbreviations: AHA= American Heart Association, c-DEV15plus= circulation-deviations 15 plus, CI= confidence interval, CPR= cardiopulmonary resuscitation, n= number of teams, NA= not available, PALS= Pediatric Advanced Life Support, s= seconds, %= percentage of teams.
